# Thermodynamics of semi-specific ligand recognition: the binding of dipeptides to the *E.coli* dipeptide binding protein DppA

**DOI:** 10.1007/s00249-021-01572-y

**Published:** 2021-10-05

**Authors:** Mohamad K. M. Zainol, Robert J. C. Linforth, Donald J. Winzor, David J. Scott

**Affiliations:** 1 School of Biosciences, University of Nottingham, Sutton Bonington Campus, LE12 5RD UK; 2 Faculty of Fisheries and Food Science, Universiti Malaysia Terengganu, Mengabang Telipot, 21030 Kuala Nerus, Terengganu Malaysia; 3 School of Chemistry and Molecular Biosciences, University of Queensland, Brisbane, QLD 4072 Australia; 4 Rutherford Appleton Laboratory, Research Complex at Harwell, Oxfordshire, OX11 0FA UK

**Keywords:** Periplasmic binding protein DppA, Dipeptide binding semi-specificity, Enthalpy–entropy compensation, Isothermal titration calorimetry, Hydrophobic interactions, Solvent structure perturbation

## Abstract

This investigation of the temperature dependence of DppA interactions with a subset of three dipeptides (AA. AF and FA) by isothermal titration calorimetry has revealed the negative heat capacity ($\Delta {C}_{p}^{o}$) that is a characteristic of hydrophobic interactions. The observation of enthalpy–entropy compensation is interpreted in terms of the increased structuring of water molecules trapped in a hydrophobic environment, the enthalpic energy gain from which is automatically countered by the entropy decrease associated with consequent loss of water structure flexibility. Specificity for dipeptides stems from appropriate spacing of designated DppA aspartate and arginine residues for electrostatic interaction with the terminal amino and carboxyl groups of a dipeptide, after which the binding pocket closes to become completely isolated from the aqueous environment. Any differences in chemical reactivity of the dipeptide sidechains are thereby modulated by their occurrence in a hydrophobic environment where changes in the structural state of entrapped water molecules give rise to the phenomenon of enthalpy–entropy compensation. The consequent minimization of differences in the value of Δ*G*^0^ for all DppA–dipeptide interactions thus provides thermodynamic insight into the biological role of DppA as a transporter of all dipeptides across the periplasmic membrane.

## Introduction

One of the still poorly understood phenomena in biology is the precise biophysical driver that leads to specific ligand recognition by a given biological macromolecule. Although we are able to characterize structures of proteins to atomic level, and are able to ascertain the fundamental thermodynamic contributions to ligand binding by a host of biophysical methods, the accurate prediction of the latter given the former is still elusive. Efforts to understand the mechanisms that regulate selective binding interaction were initially prompted by the realization that recognition and binding are universal features of all biochemical processes. One of the problems is that because many systems are tuned to bind a specific ligand, we tend to study specific binding on one system with one ligand, and then attempt to extrapolate the findings to a more general case from which we hope that broad general principles may evolve.

However, some systems are only semi-specific in that they bind a select chemical subset of ligands, whereupon the matter of interest is the source of tight ligand binding despite a lack of absolute specificity–a situation that can be addressed by studying the interactions of a diverse set of ligand types with a single acceptor system. One class of proteins that has been subjected to such study is the series of bacterial periplasmic peptide binding proteins, notably the dipeptide-binding protein (DppA) and its oligopeptide-binding counterpart (OppA).

DppA is a dipeptide-binding protein that facilitates the transport of dipeptides through the cytoplasmic membrane as well as the process of chemotaxis towards dipeptides. In keeping with the structures of other periplasmic binding proteins (Quiocho 1990; Tame et al. 1994) the ligand-binding site in DppA is located between two domains (I and III) joined by connecting strands that function as a hinge (Dunten et al. 1993; Dunten and Mowbray 1995; Bôstrom et al. 2006). In its unoccupied state the binding site is in an aqueous environment and hence accessible to dipeptide ligands. However, interaction of the binding cavity with a dipeptide heralds closure of the gap to create a closed form of DppA in which the binding site becomes isolated from the aqueous environment surrounding the protein (Dunten and Mowbray 1995). Despite differences in the extent of interactions between amino acid sidechains of DppA within the binding pocket and those of the 20 amino acids comprising potential dipeptides, the overall result is necessarily a relatively similar binding constant, or standard free energy, for all dipeptides to ensure the function of DppA as a universal active transporter of dipeptides across the periplasmic membrane.

In this investigation, isothermal titration calorimetry (ITC) is used for the thermodynamic characterization of Dpp-Adipeptide interactions. ITC affords an accurate means of quantifying biomolecular interactions (Daranas and Turnbull 2003) because of its ability to provide a full thermodynamic characterization of the molecular interaction (Ababou and Ladbury 2007). It has advantages over other biophysical techniques as it is a true solution technique that requires no substrate immobilization or labelling to allow detection of the binding interactions (Daranas and Turnbul 2003). ITC measures the dissociation constant K_d_ (and hence standard free energy ∆G^o^ as RTlnK_d_ as well as the standard enthalpy change ∆*H*º and reaction stoichiometry from a single run: the entropic contribution to the energetics (T*S*^o^) is also obtained as the difference between ∆G^o^ and ∆H^o^). The enthalpy contribution indicates the net change in heat content associated with non-covalent bond formation in the binding site (Williams and Ladbury 2004), whereas the entropy term (*T*Δ*S*º) accounts for overall changes in structural order associated with complex formation. Evaluation of those thermodynamic parameters as well as the heat capacity change (∆c^o^P) provides a means of quantifying the energetics of the protein-ligand interaction.

To obtain insight into the thermodynamics of the dipeptide-binding process isothermal titration calorimetry (ITC) has been used to characterize the interactions of DppA with three structurally similar dipeptides, namely alanine–(A–A), alanine–phenylalanine (A–F) and phenylalanine–alanine (F–A) over the temperature range 5–45 °C.

## Materials and methods

### Dipeptides

Four dipeptides comprising analine (A) and/or phenylalanine (F), namely AA, AF, FA and FF, were obtained from Sigma (Poole, Dorset, UK).

### Expression and purification of DppA

Recombinant *E. coli* DppA was expressed as previously described (Zainol et al. 2014). Concentrations were determined spectrophotometrically at 280 nm on the bases of an extinction coefficient (${A}_{1 cm}^{1\%}$) of 15.67 and a calculated molecular mass of 57.407 kDa.

### Isothermal titration calorimetry

Commercial preparations of dipeptides were dissolved in phosphate-buffered saline (1.75 mM K ${\mathrm{H}}_{2}$ PO_4_, 10 mM Na_2_HPO_4_,137 mM NaCl, 2.7 mM KCl), pH 7.4, to obtain 200 μM stock solutions of each. ITC experiments conducted in a Microcal VP-Auto apparatus (GE Healthcare). The 1.3 mL ITC cell was filled with 20 μM DppA, and the syringe with 200 μM peptide in the same buffer. Titrations were made with 5 μL aliquots, and experiments were carried out over a temperature range of 5–45 °C. The resulting heat increments were subjected to standard analysis (Wiseman et al. 1989). Reported parameter estimates refer to the mean values (± SD) from experiments performed in triplicate. The collection of ITC results at a range of temperatures also allowed evaluation of a heat capacity parameter $\Delta {C}_{P}^{o}$ (assumed temperature independent) from the slope of an essentially linear temperature dependence of $\Delta {H}^{o}$ for a given dipeptide.

## Results and discussion

**Fig. 1:**
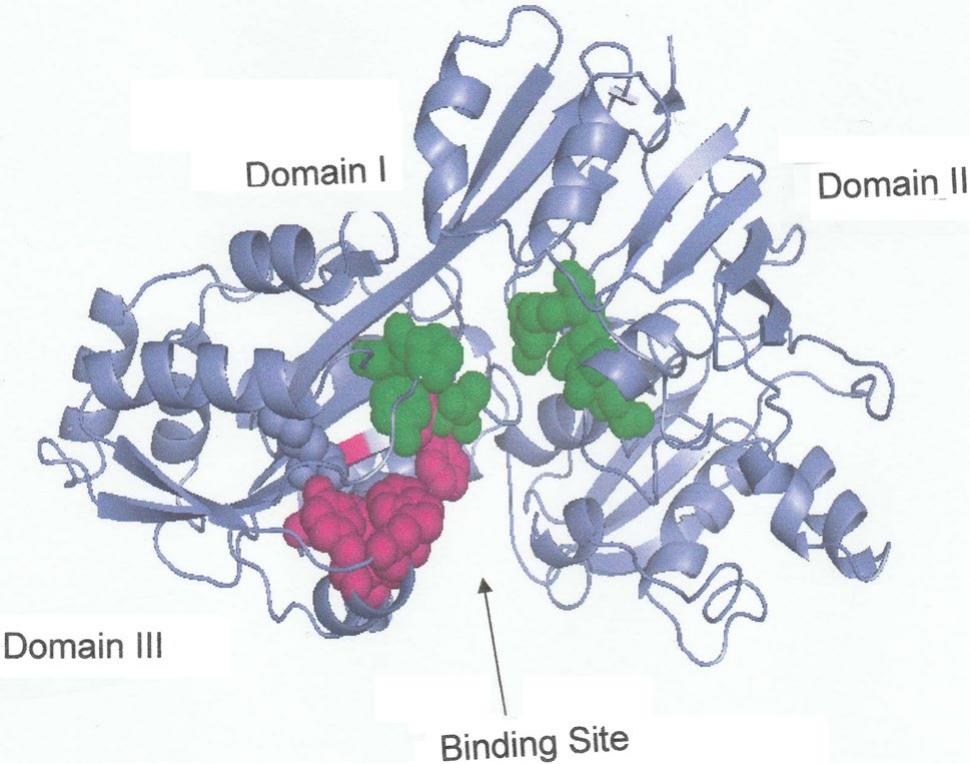
Three-dimensional structure of unliganded DppA (Dunten and Mowgray 1995) showing the location of the dipeptide-binding site in the hinge region between domains I and III. Green and red highlighting identifies the relevant amino acid residues in domains I and III respectively. (Structure constructed from coordinates listed in the Brookhaven Protein Data Bank)

**Fig. 2:**
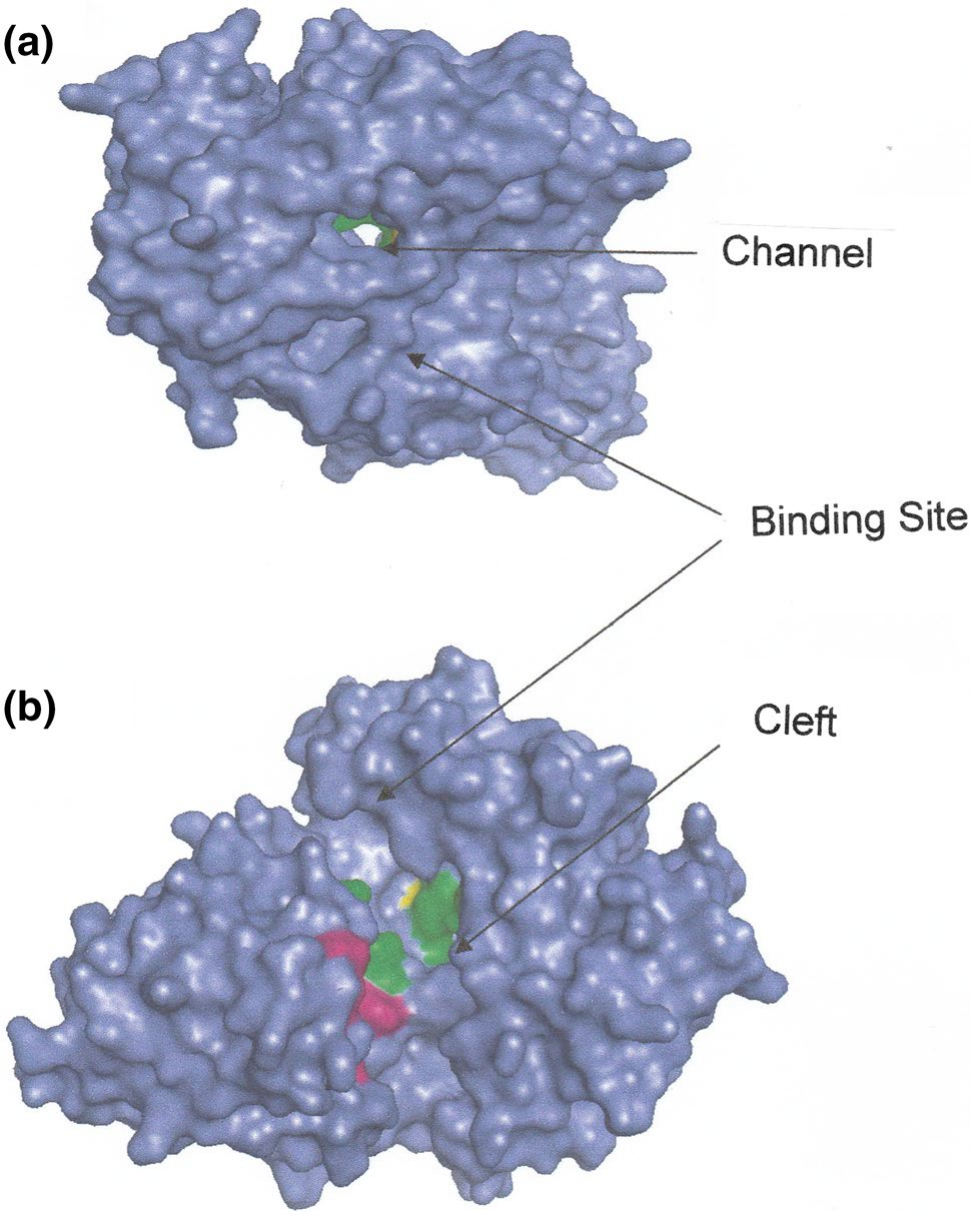
Different views of the model of unliganded DppA generated by the PyMOL program illustrating (**a**) the channel through which a dipeptide gains access to the DppA binding site, and (**b**) the binding site with relevant amino acid residues highlighted as in Fig. 1)

As in the oligopeptide binding protein OppA (Tame et al. 1994) the peptide-binding site of DppA is located between two domains (I and III), which are connected by two strands that function as a hinge region that closes upon dipeptide attachment. These features are evident from the crystal structure of unliganded DppA (Dunten and Mowbray 1995) shown in Fig. 1, where the amino acid residues comprising the binding site are highlighted in green and red in domains I and III respectively. To shed further light on the DppA–dipeptide interaction the PyMOL program (PyMOL Incentives) has been used to generate a model of unliganded DppA, two images of which are presented in Fig. 2. Reaction is initiated by passage of the dipeptide through a channel (Fig. 2a) to gain access to the binding site (participating residues highlighted as in Fig, 1), which is buried within the structure (Fig. 2b).

**Fig. 3:**
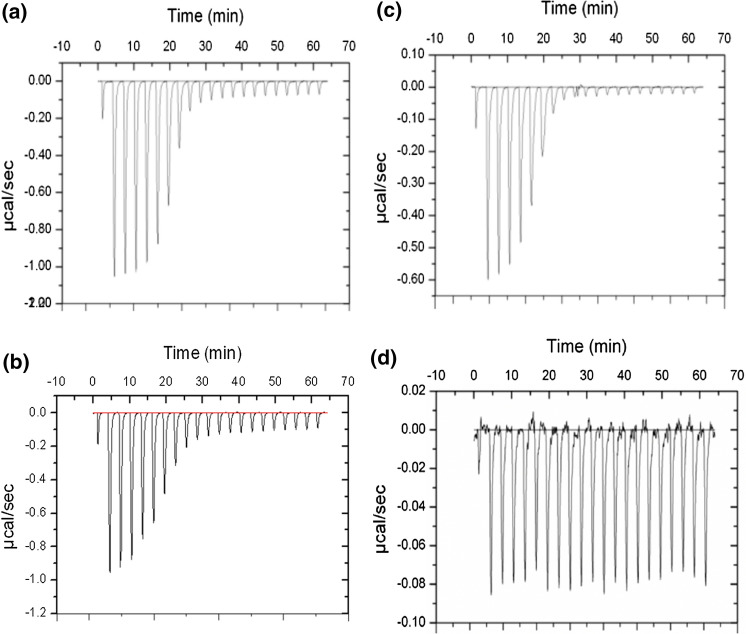
ITC traces for solutions of DppA (20 μM) supplemented with successive aliquots of dipeptide solution (200 μM). (**a**) AA; (**b**) AF; (**c**) FA; (**d**) FF

ITC traces for the stepwise addition of four dipeptides to DppA at 25 °C are presented in Fig. 3.Whereas those for AA, AF and FA (Fig. 3a–c) exhibit the titration behaviour typical of an exothermic interaction, the responses for FF were extremely small (d*Q*/dt < 0.1 μcal/s) and independent of dipeptide concentration (Fig. 3d). Failure to detect any interaction between FF and DppA is attributed to self-association of the dipeptide (via π–π stacking of the aromatic sidechains) into an oligomeric state that is too large to traverse the access channel to the buried binding site. Detailed thermodynamic characterization of the DppA–dipeptide interaction was therefore restricted to the other three peptides (AA, AF and FA).

Parameters returned by the standard analysis of isothermal calorimetry titrations (Wiseman et al. 1989) for those DppA–dipeptide mixtures at a range of temperatures are summarized in Table 1, about which the following preliminary points should be noted. In view of the well-documented instability of DppA, the return of a reasonably constant value (about 0.8) for the reaction stoichiometry (*n*) for the three systems is taken to signify 1:1 interaction between the three dipeptides and an 80% functional DppA preparation: the $\Delta {H}^{o}$ and ${K}_{d}$ values reported in Table 1 then become estimates of the standard enthalpy change and dissociation constant for the interaction between dipeptide and those functional DppA binding sites.

**Table 1:** Thermodynamic characterization of DppA–dipeptide interactions

T (K)	∆H° (kJ mol−1	Kd (nM)	*n*	∆G° (kJ mol−1)	∆H° (J K−1 mol−1)
Alanine–Alanine (A–A)					
28.15	–38 (± 2)	40 (± 4)	0.83 (± 0.03)	–39.4 (± 0.2)	+ 5 (± 8)
288.15	–47 (± 1)	34 (± 2)	0.84 (± 0.04)	–41.2 (± 0.1)	–20 (± 4)
298.15	–51 (± 3)	37 (± 10)	0.90 (± 0.03)	–42.4 (± 0.3)	–29 (± 11)
308.15	–53 (± 3)	59 (± 2)	0.77 (± 0.04)	–42.6 (± 0.1)	–34 (± 10)
318.15	–77 (± 7)	148 (± 66)	0.87 (± 0.02)	–41.6 (± 1.3)	–111 (± 26)
Alanine–Phenylalanine (A–F)					
278.15	–38 (± 4)	356 (± 20)	0.80 (± 0.02)	–34.3 (± 0.1)	–13 (± 15)
288.15	–42 (± 3)	354 (± 19)	0.80 (± 0.03)	–35.6 (± 0.1)	–22 (± 11)
298.15	–67 (± 7)	357 (± 26)	0.83 (± 0.04)	–36.8 (± 0.2)	–101 (± 24)
308.15	–70 (± 4)	552 (± 65)	0.80 (± 0.05)	–36.9 (± 0.3)	–107 (± 14)
318.15	–72 (± 2)	642 (± 40)	0.80 (± 0.07)	–37.7 (± 0.1)	–108 (± 7)
Phenylalanine–Alanine (F–A)					
278.15	–27 (± 4)	211 (± 7)	0.90 (± 0.02)	–35.5 (± 0.1)	+ 30 (± 15)
288.15	–30 (± 6)	203 (± 23)	0.75 (± 0.06)	–36.9 (± 0.3)	+ 24 (± 22)
298.15	–39 (± 7)	227 (± 21)	0.84 (± 0.03)	–37.9 (± 0.3)	–4 (± 25)
308.15	–43 (± 11)	244 (± 39)	0.91 (± 0.02)	–39.0 (± 0.5)	–13 (± 37)
318.15	–44 (± 8)	293 (± 17)	0.84 (± 0.05)	–39.8 (± 0.3)	–13 (± 26)

**Fig. 4:**
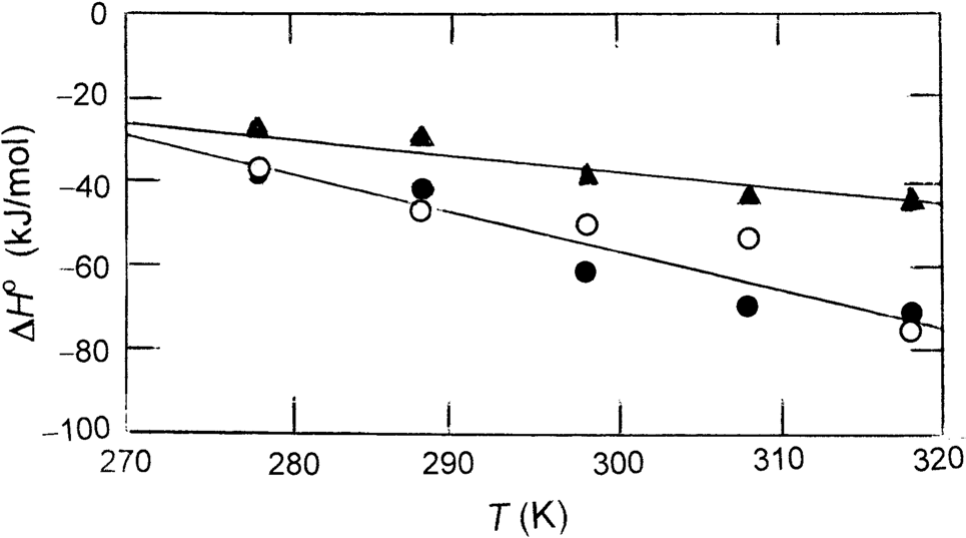
Estimation of the heat capacities ($\Delta {C}_{p}^{o}$) from essentially linear temperature dependences of Δ*H*^o^ for the interactions of DppA with dipeptides AA (●), AF (◯) and FA (▲)

In terms of heat release or uptake the DppA–dipeptide mixtures are all exothermic systems that can be described thermodynamically by negative changes in standard enthalpy ($\Delta {H}^{o}$) as well as negative estimates of heat capacities Δ*C*_*P*_, taken as the slopes of essentially linear temperature dependences of $\Delta {H}^{o}$ (Fig. 4). Those heat capacity changes for the AA (◯) and AF (●) ligands were similar ($\Delta {C}_{p}^{o}\approx$−900 J ${\mathrm{mol}}^{-1} {\mathrm{K}}^{-1}$), whereas that for FA (▲),–470 J mol^−1^ K^−1^, was approximately half that value. As noted by Lumry and Rajender (1970) as well as by Singer (1977), it is far more rewarding from a molecular viewpoint to compare $\Delta {H}^{o}$ values for these highly hydrophobic systems with their standard free energy counterparts ($\Delta {G}^{o}$) that are calculated from the dissociation constant ($\Delta {G}^{o}=RT \mathrm{ln}{ K}_{d}).$ From column 5 of Table 1 it is evident that those standard free energy changes show much less dependence on temperature than that exhibited by $\Delta {H}^{o}$ because of compensating changes in the standard entropy change $\Delta {S}^{o}$ (final column of Table 1).

**Fig. 5:**
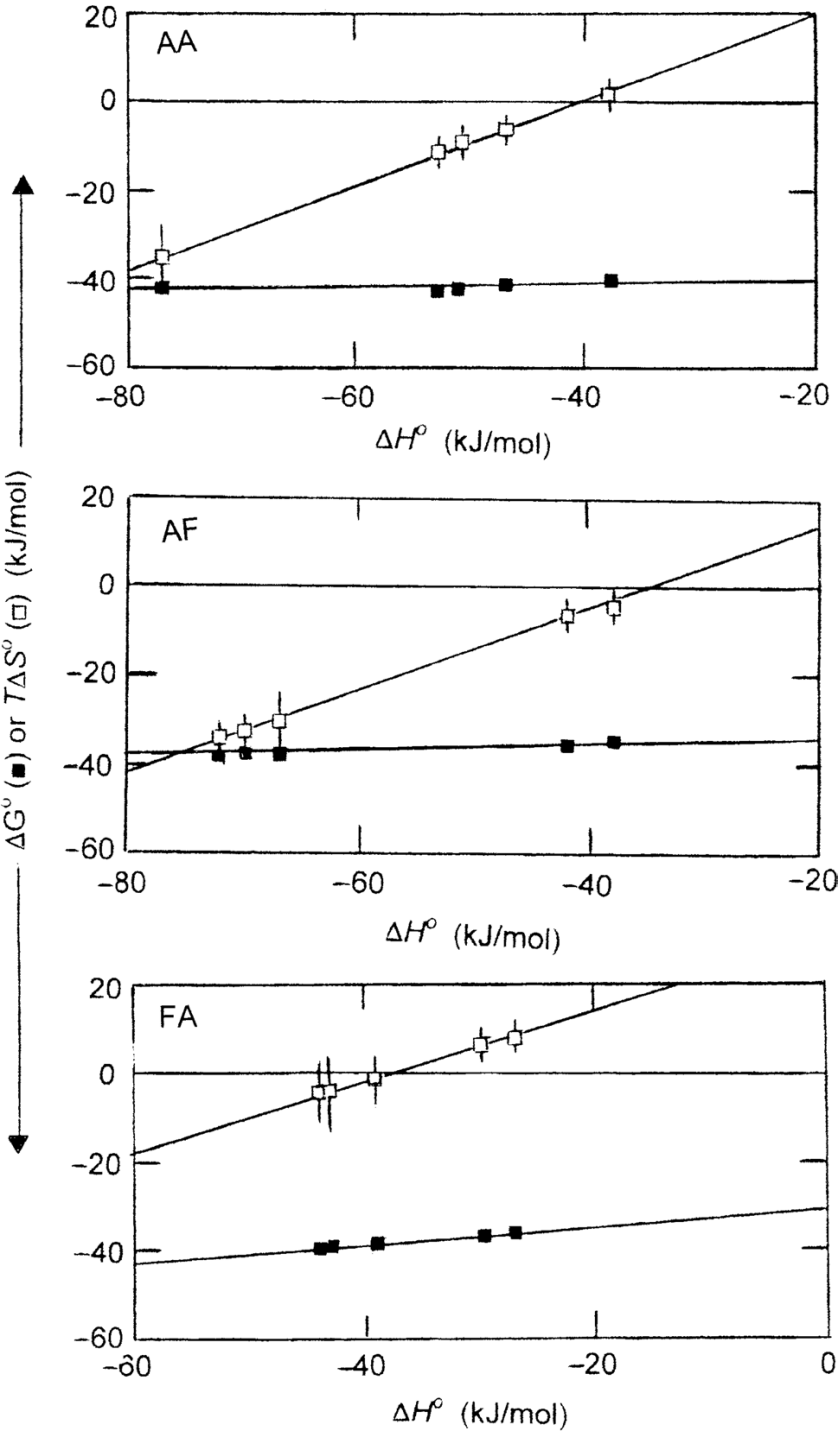
Demonstration of the extents of enthalpy–entropy compensation in the interactions of the dipeptides A–A, A–F and F–A with the periplasmic dipeptide-binding protein DppA. The format has been chosen to highlight not only the relative insensitivity of Δ*G*^o^ to changes in Δ*H*^o^ but also the corresponding variations in the entropic contribution (*T*Δ*S*^o^) that counter the enthalpic driving force of these exothermic reactions

The extent of the compensatory effect is emphasized in Fig. 5, which compares the dependencies of $\Delta {G}^{o}$ and *T* $\Delta {S}^{o}$ on $\Delta {H}^{o}$. For the DppA interaction with AA the enthalpy–entropy compensation is extreme in that $\Delta {G}^{o}$ is essentially independent of $\Delta {H}^{o}$ [slope = 0.04 (± 0.14)], whereas the corresponding slope of the *T* $\Delta {S}^{o}$−$\Delta {H}^{o}$ dependence is 0.96 (± 0.14). A similar situation applies to the DppA interaction with AF in that the two slopes are 0.08 (± 09) and 0.92 (± 09) respectively. For the FA interaction there is only partial enthalpy–entropy compensation in that the dependence of $\Delta {G}^{o}$ upon $\Delta {H}^{o}$ is characterized by a larger slope of 0.21 (± 0.10), while that for *T* $\Delta {S}^{o}$ is correspondingly smaller [0.79 (± 0.12)].

A striking feature of the enthalpy–entropy compensation observed in Fig. 5 for the DppA–dipeptide interactions is the size of the unfavourable entropic contribution (*T*$\Delta {S}^{o}$) to the driving force of the reaction (Δ${G}^{o}$). Although some loss of protein conformational entropy necessarily accompanies closure of the binding-pocket hinge, that phenomenon alone is unlikely to generate a *T*$\Delta {S}^{o}$ contribution of that magnitude. Instead, the results all find rational explanation in terms of the energy contributions from changes in solvent structure that play such an important role in hydrophobic and electrostatic interactions (Kauzman, 1959; Lumry and Rajender 1970; Privalov and Gill, 1988, Grundwald and Steel 1995; Breiten et al. 2013; Dragan et al. 2017; Scott et al. 2019).

For a reaction characterized by temperature dependence of the standard enthalpy change the corresponding temperature dependence of the dissociation constant assumes the form (Naghibi et al. 1995)

1 $$Ln\left( {K_{D} } \right)_{0} = LnK_{D} + \frac{{\left( {\left( {\Delta H_{0} } \right) - T_{0} \Delta C_{P} } \right)}}{R}\left[ {\frac{1}{{T_{o} }} - \frac{1}{T}} \right] + \frac{{\Delta C_{P} }}{R}Ln\left[ {\frac{1}{{T_{o} }}} \right]$$

where zero subscripts on *K*_*D*_ and *T* denote values at a selected reference temperature. On the grounds that the temperature insensitivity of *K*_*D*_ for AA and AF seemingly signifies a value of essentially zero for Δ*C*_*P*_ for the interaction between DppA and dipeptide, concern has been expressed that isothermal titration calorimetry is detecting additional associative (enthalpic) forces that are not involved in protein–dipeptide complex formation being monitored (Naghibi et al. 1995; Liu and Sturtevant 1995, 1997; Mizoue and Tellinghuissen 2004).

Temperature-dependent changes in water structure are the logical source of that additional enthalpy change (Walrafen et al. 1986a, b; Lumry 2003a, b; Smith et al. 2004, 2005; Wernet et al. 2004). In the present context, an enthalpic energy contribution is involved in generation of the enhanced structural state of water molecules that are trapped within the hydrophobic loop containing complexed dipeptide (Dunten and Mowbray 1995). At the lowest temperature (278 K) very little additional associative force is required because of the highly structured state of water in the vicinity of its density maximum (277 K). At higher temperatures, the increased flexibility of water structure mandates the input of additional enthalpic energy to maintain the highly structured state of water molecules located in a hydrophobic environment; and thereby gives rise to a temperature-dependent decrease in Δ*H*^*o*^ (the observed heat capacity effect). However, that increase in associative force (favourable enthalpic energy) is at the expense of its *T*Δ*S*^*o*^ counterpart because the strengthening of hydrogen bonding between water molecules impacts adversely on water flexibility. A consequence of this enthalpy–entropy compensation is that perturbations of water structure bring about no change in standard free energy of the system. Therein lies a very plausible explanation for the observation of a Δ*C*_*P*_ effect but no change in Δ*G*^*o*^ for hydrophobic interactions. It also dispels the above-mentioned concern that calorimetry measurements may reflect an additional associative force that is not involved in the protein–ligand interaction upon which the quantitative expression (Wiseman et al. 1989 for the determination of Δ*H*^*o*^ and *K*_d_) is based. Rather than signifying that Δ*C*_*P*_ ≈ 0 for complex formation, the insensitivity of *K*_*d*_ to temperature variation is reflecting the existence of enthalpy–entropy compensation in the solvent-structure perturbation responsible for the enthalpy contribution to Δ*G*^o^ arising from the presence of structured water within the DppA–dipeptide complex.

Additional evidence for the existence of a link between enthalpy–entropy compensation and the perturbation of solvent structure has been provided by a study of urea dimerization, which affords a much simpler and more dramatic example in that the compensation by the standard entropy term leads to a slightly positive standard free energy change (Δ*G*^0^ =  + 0.066 molal^−1^) despite a sizeable negative enthalpic contribution to Δ*G*^0^ (Winzor and Wills 2019). Furthermore, that large negative entropic contribution to the energetics of urea dimerization correlates with a quantum-mechanics-based chemical structure (Ramondo et al. 2007) showing the adverse effects of changes in water structure on dimer formation. Indeed, much of the negative enthalpy contribution to Δ*G*^0^ stems from a hydroxyl–carbonyl hydrogen bond as well as hydrogen bonding between water molecules in a structured shell surrounding the urea molecules. The days of regarding water as an inert solvent in protein solutions must clearly cease.

**Fig. 6:**
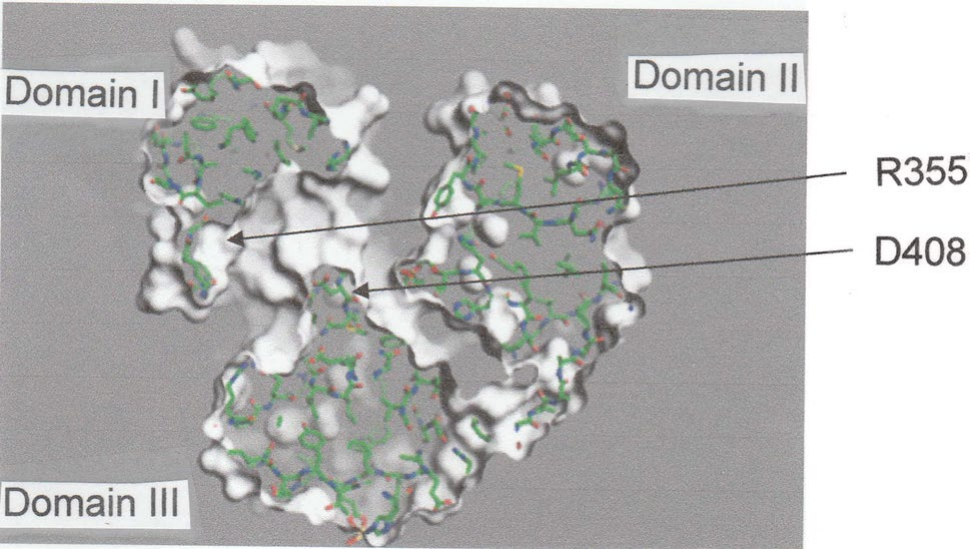
A slice through a model of the unliganded DppA structure with the polypeptide chain superimposed in green that pinpoints the binding site residues to which the α-amino and α-carboxyl groups of a dipeptide attach (D408 and R355 respectively)

After that diversion to rationalize the concurrent phenomena of negative heat capacity and enthalpy–entropy compensation in hydrophobic interactions, we now return to specific consideration of the present findings for the three DppA–dipeptide systems examined. As mentioned above, the dipeptide-binding site is located between domains I and III of DppA (Figs. 1, 2}. Further detail is provided in Fig. 6, which shows a slice through the unliganded DppA structure with the polypeptide chain interposed. The amino terminus of the dipeptide reacts with an aspartate residue (D408) in Domain III, beside which is a loop of hydrophobic residues that creates a pocket to accommodate the N-terminal amino acid residue. Another pocket adjacent to a DppA arginine residue (R355) in Domain I serves the same purpose for the dipeptide C-terminal amino acid residue (Denton and Mowbray 1995; Nickitenko et al. 1995). This loop is also mainly hydrophobic but includes aspartate (D153) and lysine (K498) residues. As noted in the Introduction, the binding of a dipeptide triggers closure of the hinge to generate the closed form of DppA in which the binding pocket is isolated from the aqueous phase surrounding the protein. Although most water molecules are expelled from the binding pocket by that hinge closure (Williams and Ladbury 2004), some remain entrapped in the newly created hydrophobic environment–particularly in the two loops accommodating the dipeptide sidechains. The extent of the entropy decrease and the enthalpy enhancement effected by this entrapment of water molecules in highly structured states thus becomes dependent upon their number.

Inspection of Table 1 shows that AA binds to DppA much tighter than the other two dipeptides (AF and FA) in which a larger aromatic phenylalanine residue replaces one of the alanine residues. As noted by Dunten and Mowbray (1995), solvent occupies the excess space surrounding the dipeptide sidechains in both DppA loops, which would therefore contain more structured water molecules when the entrapped sidechain is small. The largest decreases in entropy, enthalpy and hence ${K}_{d}$ are thus the predicted outcomes for the binding of AA to DppA. Although that rationale accounts for the smaller ${K}_{d}$ observed for the interaction of DppA with AA, the application of the same logic to account for the difference in dissociation constants effected by substitution of phenylalanine for the N-terminal and C-terminal dipeptide alanine residues implies that fewer water molecules are retained when F is the C-terminal residue. Inasmuch as the DppA loop surrounding the C-terminal residue is smaller than its N-terminal counterpart there is certainly potential for water molecules to be displaced by the replacement of A by F–particularly in the event that there is any structural rearrangement of the loop as the result of π-stacking of aromatic rings. Any such structural perturbation within the binding pocket could also affect the alignment of the dipeptide anchored by electrostatic interactions of its carboxyl and amino terminals with DppA residues R355 and D408 respectively; and hence alter the strength of the hydrogen bonding that occurs between DppA and the backbone carbonyl and amino groups of the dipeptide (Dunten and Mowbray 1995).

This ITC study of the DppA interaction with a subset of dipeptides (AA, AF and FA) has certainly detected differences in the thermodynamic parameters for the three dipeptides. However, the enhanced enthalpic contribution ($\Delta {H}^{o})$ to the energetics effected by an increase in temperature is largely offset by the entropy contribution (*T* $\Delta {S}^{o}$) to yield a standard free energy change ($\Delta {G}^{o}$) that varies little with temperature. Furthermore, the differences between $\Delta {G}^{o}$ for the three systems at a given temperature are sufficiently small to be compatible with the functional role of DppA as a transporter of all three peptides despite their different composition and/or sequences. In other words the interactions of the N-terminal amino and C-terminal carboxyl groups with DppA residues D408 and R355 respectively ensures a specificity for dipeptides; and any additional consequences of DppA–sidechain interactions are modulated by their occurrence in hydrophobic loops where changes in the structural state of entrapped water molecules give rise to the phenomenon of enthalpy–entropy compensation. Therein lies a simple explanation of the enthalpy–entropy compensation observed in the interaction of DppA with a range of dipeptides at 25 °C (Payne et al. 2000); and also the effectiveness of DppA as a general facilitator of dipeptide transport through the cytoplasmic membrane.

## Concluding remarks

The present thermodynamic investigation of the temperature dependence of DppA interactions with a subset of three dipeptides (AA, AF and FA) by isothermal titration calorimetry has coupled the negative heat capacity (Δ*C*_*P*_) that is characteristic of hydrophobic interactions with the perturbation of solvent structure that is responsible for the observed enthalpy–entropy compensation. That general explanation has then been reinforced by considering the thermodynamic findings for the present system in terms of the X-ray crystallographic structure for the complex between DppA and the dipeptide GL (Dunten and Mowbray 1995). Specificity for dipeptides stems from appropriate spacing of designated DppA aspartate and arginine residues for electrostatic interactions with the terminal α-amino and α-carboxyl groups of the dipeptide, after which the binding pocket closes to become completely isolated from the external aqueous environment. Thereafter any differences in chemical reactivity of the dipeptide sidechains are nullified by their location in two loops of the DppA sequence, that contain trapped water molecules in a highly structured state because of the hydrophobic environment, thereby rendering the strength of binding (Δ${G}^{o}$) essentially the same for any dipeptide. Indeed, changes in the structural state of water may well dominate the magnitudes of Δ${H}^{o}$, *T*Δ${S}^{o}$ and hence Δ${G}^{o}$ for the system. Enhancement of the structural state of water by a strengthening of hydrogen bonding between water molecules necessarily entails a loss of randomness (a decrease in entropy) that counters the enthalpic gain and hence moderates the change in standard free energy Δ${G}^{o}$ (Lumry and Rajender 1970; Sturtevant 1977; Privalov and Gill 1988). The explanation of enthalpy–entropy compensation in terms of perturbations of water structure is slowly but surely gaining ground (Dragan et al. 2017; Scott et al. 2019).
